# A Parent-Mediated Intervention for Newborns at Familial Likelihood of Autism: Initial Feasibility Study in the General Population

**DOI:** 10.1007/s41252-022-00262-w

**Published:** 2022-06-09

**Authors:** Dominique B. Cleary, Angela Bunney, Lindy Henry, Michelle Renton, Joanna Granich, Jonathan Green, Murray T. Maybery, Andrew J. O. Whitehouse

**Affiliations:** 1 Telethon Kids Institute, The University of Western Australia, 100 Roberts Road, Subiaco, Western Australia 6008 Australia; 2 School of Psychological Science, The University of Western Australia, Perth, Australia; 3 Division of Neuroscience & Experimental Psychology, School of Biological Sciences, University of Manchester, Manchester, UK; 4 Manchester Academic Health Science Centre, Manchester University NHS Foundation Trust, Greater Manchester Mental Health NHS Trust, Manchester, UK

**Keywords:** Autism, Intervention, Prodromal, Infancy, Parent-child interaction, ORIGINS sub-project

## Abstract

**Objectives:**

Developmental theory and previous studies support the potential value of prodromal interventions for infants at elevated likelihood of developing autism. Past research has supported the efficacy of parent-mediated prodromal therapies with infants from as early as 7 months. We outline the rationale for implementing interventions following this model from even earlier in development and report on the feasibility of a novel intervention developed following this model of parent-mediated infant interventions.

**Methods:**

We report a feasibility study (*n* = 13) of a parent-mediated, video-aided intervention, beginning during pregnancy, focussed on parent-infant interactions. The study evaluated the feasibility of this intervention initially with a general population sample. Feasibility was assessed across four domains (acceptability, implementation, practicality and integration) using self-report questionnaire, semi-structured interviews with parents and therapists, attendance and assessment completion.

**Results:**

Feasibility assessment shows that the intervention was acceptable, with all participants reporting that they had benefited from the program, with perceived positive benefits to their understanding of and communication with their infant, and that they had integrated program teachings into everyday life. The intervention was implemented as planned with 100% attendance for the core sessions. Changes to minimise the number of antenatal sessions was suggested to improve practicality.

**Conclusions:**

This study found initial feasibility for this intervention in a general population sample. This suggests parent-mediated video feedback interventions are a promising format to be implemented within the perinatal developmental time period.

Autism spectrum conditions (hereafter autism) are neurodevelopmental conditions characterised by difficulties in social behaviour and communication, and restricted and repetitive behaviours, with the level of support required by an autistic individual varying broadly from requiring some support, to requiring very substantial support (American Psychiatric Association, 2013). Currently, the mean age of diagnosis of autism in Australia is 4 years (Bent et al., 2015), although reliable diagnoses can be made from the second year of life (Guthrie et al., 2013; Pierce et al., 2019), with diagnosis relying on the presentation and identification of key behavioural symptoms.

Early diagnosis and early intervention are hypothesised to improve long-term outcomes (Johnson et al., 2007; Rutter, 2006). This promise of early intervention has led to a strong research focus on identifying the children likely to receive an autism diagnosis as early as possible (Jones et al., 2014; Tanner & Dounavi, 2021; Zwaigenbaum et al., 2015), often examining the development of infant siblings of children diagnosed with autism, who have an elevated likelihood of later being diagnosed with autism themselves (Ozonoff et al., 2011). Growing evidence indicates the importance of infant neurodevelopment in the developmental cascade towards autistic symptoms (Jones et al., 2014), a developmental period not frequently addressed in interventions, likely because diagnoses typically occur later in childhood. In line with the recognition of early developmental differences, converging evidence has supported the possibility that implementation of therapies at an earlier age, prior to the emergence of the diagnostic behaviours of autism, could significantly alter the developmental trajectory of autistic symptom presentation and impact long-term outcomes.

Research from the developmental sciences has shown that while human infants are born with a limited behavioural repertoire (Nagy, 2011), rapid postnatal development occurs as infants transition from early reflexes to volitional behaviours at 6–8 weeks of age (Shultz et al., 2018). These developments are, however, contingent on the provision of input from a caregiver (Gibson, 1988), and these interactions within the parent-infant dyad have been posited as crucial in early development (Feldman, 2007; Goldberg, 1977). The “interactive specialisation” (IS) model (Johnson, 2000) of developmental cognitive neuroscience proposes that the “social brain” develops through an active process of postnatal interaction with the environment. The IS model hypothesises gradual changes in response to postnatal exposures in specialisation—the degree to which the responses of a cortical region are tuned to a certain class of input—and in localisation—the spatial extent of cortex activated following a stimulus presentation. This model complements evidence within developmental psychology around the importance of the early social environment on later social and communicative functioning, and on how the early environment can be disrupted in atypical development.

In the context of autism, there is good evidence that infants later diagnosed with the condition have reduced or impaired function in one or more underlying social biasing mechanisms early in life such as response to eye gaze being disrupted by 6 months (Elsabbagh et al., 2012) and as early as 2 months of age (Jones & Klin, 2013), and disrupted response to speech sounds from as early as 4 months (Lloyd-Fox et al., 2013). While the evidence is still emerging for the time periods around which these changes occur—with some mechanisms appearing to be intact at birth, such as eye gaze (Jones & Klin, 2013)—it is clear that within the first year of life social biasing mechanisms are impacted.

It is further suggested that this disruption in social orienting among infants later diagnosed with autism can lead to differences in parent-infant interaction styles. For example, autistic children present fewer leads for communication partners (such as parents) to follow, and these interactional leads are often less clearly understood and are more easily missed (Adamson et al., 2010). Caregivers may attempt to compensate for their child’s limited contribution by directing the child’s attention rather than following the child’s focus or actions to support joint attention (Freeman & Kasari, 2013; Wan et al., 2012). This resulting increase in directive parental style may further limit opportunities for autistic children to improve their communicative skills. Therefore, to optimise development for autistic children, a higher proportion of sensitive and supportive partner responses may be required for optimal growth in communication skills (Adamson et al., 2010; McDuffie & Yoder, 2010; Ruble et al., 2008). This hypothesis has been supported by research showing that parenting style is linked to later developmental outcomes, with interaction styles that are sensitive and responsive to child cues associated with favourable long-term social and communicative outcomes for children (Siller & Sigman, 2002; Tamis-LeMonda et al., 2001).

These findings raised the possibility that intervening in parent-child interactions during this early period may address critical time-bound developmental transitions in vulnerable infants and promote the development of longer-term outcomes. Following this theoretical premise, an intervention focussed on mediating parent-infant interaction has recently been trialled (Green et al., 2017; Whitehouse et al., 2019). This intervention implemented an adaptation of the Video Interaction for promoting Positive Parenting (VIPP) programme (Juffer et al., 2008), modified to target, from as early as 7 months, the autism prodrome in infants at elevated familial likelihood of developing autism. This intervention (iBASIS-VIPP) involved the filming of care-giver-infant interactions during individual sessions, which provided the basis for video feedback discussion focussed around the social-communicative aspects of each parent-infant dyad. The therapist assisted with the framing of observations, caregiver self-reflection, identifying positive examples of infant behaviours and responsive caregiver interactions, and focusing on change in the caregiver’s communicative responses to the infant. Green and colleagues’ iBASIS randomised controlled trial (RCT) tested the impact on infants with an older autistic sibling of enhancing parent-infant interactions through video feedback (*n* = 28) compared to a no-intervention control condition (*n* = 26). Commencing when the infants were 7–10 months old, results suggested significant improvements in parental non-directiveness at the immediate treatment endpoint (at 15 months) (Green et al., 2015). Follow-up conducted 2 years later showed sustained treatment effect in improving parent-infant social interaction as well as reducing emergent autism symptom severity (Green et al., 2017).

A further RCT (the AICES study; Whitehouse et al., 2019) tested the same parent-mediated intervention (iBASIS-VIPP), in an indicated sample of clinically referred infants showing early behavioural signs associated with later autism (mean age 12 months). Infants receiving iBASIS-VIPP (*n* = 50) were compared to infants receiving intervention as usual (*n* = 53). Assessment post-treatment (at 18 months) showed significantly greater improvements in parent-reported (but not researcher-administered) language and communication outcomes for infants receiving the intervention (Whitehouse et al., 2019). Follow-up at 3 years demonstrated a sustained benefit in autism symptom severity evident in the intervention group, with a significantly reduced likelihood of receiving an autism diagnosis (Whitehouse et al., 2021).

Whilst these studies provide replicated support for the sustained efficacy of prodromal interventions for autism from as early as 7 months of age on social development, the possibility of even earlier intervention has yet to be investigated. Research into infant development in the first months of life provides a compelling rationale for considering interventions to commence as early as birth. Shultz et al. (2018) provide a review of early development in the first 6 months, with a focus on the behaviour and neurodevelopment involved in infants’ developing orientation towards social stimuli. They review a series of “pivotal transitions” in the first 6 months of typical infancy, turning points that indicate a shift from initial reflex-like responses into contingent social actions, and the impact when these developmental transitions are atypical, such as for infants later diagnosed with autism. These transitions that provide an orientation towards social stimuli in typical development are hypothesised to provide the developmental groundwork to support later social development.

One of these is the development of eye gaze. At birth, infants display initial reflexive visual orienting to certain social stimuli, such as faces, supported by the subcortical visual system (Johnson et al., 1991). This initial reflex appears to decline at 4–6 weeks of age, as transitions in cortical circuits develop and inhibit these reflexive behaviours. A transition to more volitional visual orienting is then seen at 2 months of age, indicating the development of volitional social biasing behaviours is underpinned by rapidly maturing cortical pathways (Johnson et al., 2015). Other newborn biases, such as the orientation to auditory stimuli (Field et al., 1980), follow a similar transition across this early developmental period.

Prospective studies have explored these developmental transitions in infants with an elevated familial likelihood of developing autism. A key study examined eye-fixations in young infants who were later diagnosed with autism compared with typically developing infants (Jones & Klin, 2013). At 2 months of age, levels of eye-fixations were relatively high in the autism group compared to the typically developing group; however, while eye fixations increased in the typically developing group from 2-8 months of age, they decreased during this period in the autism group. Similarly, a recent study found that infant siblings of autistic children were delayed in the increasing attention to visual social information between 2 and 3 months of age seen in infants with no family history of autism (Bradshaw et al., 2020). The level of social attention observed at this early stage was also found to be positively associated with social communication and nonverbal cognitive skills at 12 months. These data indicate that the developmental processes underlying social attention may already be different at 2 months of age in children later diagnosed with autism, indicating an important period of development between 2 and 3 months of age when infants vulnerable to developing autism do not display the same biases to attend to visual social stimuli.

Taken together with findings indicating critical transitions in the development of social orienting in the general population in the first 3 months of life, this research has led to the hypothesis that disruptions in the transition from reflexive (subcortical) social attention to preferential (cortical) social attention may be the early origins of social differences that are the hallmark of autism (Shultz et al., 2018). It therefore follows that these early months may reflect a very early sensitive developmental period during which to target already proven treatment modalities. These considerations raise the possibility that improving the quality of parent-infant interactions from the first moments of life may convey longer-term benefits in the social development of the infant and that this may have implications for how we provide clinical management to infants at elevated likelihood for autism (such as those with a family history).

While establishing the feasibility and efficacy of interventions with infants with an elevated likelihood of autism is central, there is established precedence that this form of intervention would also be appropriate, and potentially beneficial, to typically developing infants. iBASIS-VIPP was adapted from the “Video Interaction to promote Positive Parenting” (VIPP) model initially developed in the context of neurotypical infants at environmental risk (Juffer et al., 2008; Moss et al., 2011). The VIPP model has been successfully adapted and found acceptable for use within the first 6 months of infancy with mothers with postnatal psychiatric disorders and their infants (Stein, et al., 2006). In the Green et al. (2017) trial, the iBASIS-VIPP intervention was used successfully without adverse effects reported and showing sustained social gains in a context in which about 50% of the infants turned out to be neurotypical, suggesting the feasibility and effectiveness of this parent-mediated video feedback model with even non-clinical community populations. While the eventual intention of this intervention project therefore is to optimally support the development of newborns and their families with higher than usual likelihood of autism, the ethical context for this work is that the majority of infants from such families will actually have neurotypical development.

We determined therefore that as a first step it is essential that this intervention proves its feasibility with families at no elevated risk in the community population. This study takes the novel step of applying the model of parent-infant intervention to the perinatal period and over the first few months of life, with the aim of assessing the feasibility of this intervention. As such, the core outcomes examined were measures of feasibility, assessed across four domains of the framework for conceptualising feasibility studies developed by Bowen et al. (2009): acceptability, implementation, practicality and integration. We hypothesised that a model of parent-infant intervention shown to be feasible and effective in later stages of infancy will be feasible to implement as a perinatal intervention. While child measures were also collected to assess for the feasibility of measurement, outcome analysis will not be presented since efficacy estimation was not the intention of this feasibility study.

## Methods

### Participants

This study used an observational design to examine the feasibility of the intervention program with a sample of families (*n* = 13). For this study, the intervention program was trialled with families from the general community, with minimal exclusion criteria. All participants were recruited through the ORIGINS Project, a longitudinal birth cohort study operating out of Joondalup Health Campus, Perth, Western Australia (Silva et al., 2020). Recruitment took place through private and public obstetric and antenatal clinics at Joondalup Health Campus, where all women who presented during early pregnancy (~ 8–22 weeks of pregnancy) were informed of the opportunity to participate in ORIGINS and were provided with recruitment material. Following enrolment in ORIGINS, women were then provided with information about sub-studies for which they were eligible, including the current study.

Thirteen pregnant women were recruited for the current study. Inclusion criteria were that the pregnancy was a primiparas singleton pregnancy, that English was the main language spoken at home and that the family were intending to remain in the Perth metropolitan area for the following 2 years. Participants were excluded from the study if the developing foetus had been diagnosed with a serious medical condition requiring ongoing care. One family recruited into the study was determined to be pregnant with twins after their recruitment. This family was retained in the study, with data collected for one infant.

#### Demographics

For all families, the household included two parents, the elected primary caregiver was the biological mother and the infant was the first child. Elected caregivers had a relatively high level of education, all having completed a minimum 12 years of schooling and eight of the women holding a tertiary qualification (two held a vocational certificate or diploma and six held a university degree). All families reported household incomes above average, with all but one family reporting an annual household income above $AU100,000 a year, where the average Western Australian metropolitan household income was estimated at $AU82,940 based on 2016 census data (Australian Bureau of Statistics, 2016). Ethnicity was reported for primary caregivers, with parents identifying as being of ethnic extraction as follows: Australian Peoples (1), British (7), Eastern European (1), Mainland South-East Asian (1), Southern Asian (1), Western European (1), missing (1).

### Procedures

Families received two antenatal sessions, followed by 7–10 postnatal sessions (7 core sessions and optional “booster” sessions) run by clinically qualified Child Health Nurses who had undergone specific training on the intervention. Sessions occurred within a home setting, with core sessions occurring initially fortnightly, then monthly, up to when the baby was 5 months old, with booster sessions offered from 6 to 8 months. Participating families elected a primary caregiver who was available for all sessions, and outcome measures were assessed with this caregiver. Secondary caregivers were able to attend sessions and encouraged to do so. All intervention sessions were videotaped and a random subset of 5% of intervention sessions (spread across each session and clinician) were rated on a fidelity rating scale, conducted by an independent assessor who was one of the developers of the program. They were assessed on general therapeutic procedures including content of the session in line with manual (covering review of previous sessions, introduction of session theme, illustration of session theme through video review, review of the session), working with the parent (eliciting parent feedback, structuring feedback to deliver to the parent and managing parent focus, as well as appropriate pacing of the session) and interpersonal effectiveness (sensitivity to and validation of the parent).

Initial assessments of the pre-natal sessions of the program were collected at 1 month of age at the first postnatal session with families (time 1), and post-program assessments took place at 6–8 months (time 2). Infants and parents were assessed to determine the feasibility of the intervention, including acceptability to families (time 1 and 2), and the feasibility of assessing behavioural measures of the infants’ development and interaction skills (time 2 only). Therapist assessment of feasibility was also collected at this time point.

#### Intervention

The current pilot study builds on the logic of the iBASIS-VIPP intervention tested in the previous AICES and iBASIS studies (Green et al., 2017; Whitehouse et al., 2021) and is designed as a parent-mediated infant intervention utilising video feedback. The protocol piloted here builds on themes from those trials and seeks to improve parents’ ability to identify their newborn’s communication cues and increase their sensitivity and responsiveness to these cues during social interactions, which is hypothesised to promote social communication skill development and reduce the severity of autistic symptoms. The intervention protocol applies a logic established and evidenced from previous prodromal and post-diagnostic interventions, which indicate that altering an infant’s reciprocal social environment with caregivers in purposive ways through therapy will impact positively on the cascade of social development and improve child outcomes (e.g. Green et al., 2017; Whitehouse et al., 2019). Table 1 provides an overview of the content included in the intervention session.

**Table 1:** An overview of the intervention sessions

	Name	Individual/group	Baby’s age	Location	Duration	Theoretical focus
Antenatal						
1	Introduction	Small group	Third trimester	Clinic	1.5 h	Introduction to the program, brain development and an overview of babies’ social behaviours.
2	Preparing for connection	Small group	Third trimester	Clinic	1.5 h	Newborn communication: Psychoeducation about a newborn’s state of alertness and cues, and responding sensitively.
Postnatal						
3	Baby delight	Individual	4 weeks	Home	1 h	*Enjoying your baby*: Use of the NBO* to explore and delight with the parent in their baby’s unique capacities.
4	Baby discovery	Individual	6 weeks	Home	1 h	Strengthening parent-child relationship through observation of babies as intentional beings, and understanding babies take time to process experiences.
5	Baby responding	Individual	8 weeks	Home	1 h	Enhancing parents understanding of interaction skills—voice and gentle touch.
6	Baby play	Individual	10 weeks	Home	1 h	Joint engagement and contingent responses.
7	Baby matching	Individual	12 weeks	Home	1 h	Affect and facial imitation.
8	Baby talk	Individual	16 weeks	Home	1 h	Verbal and motor imitation.
9	Baby explorer	Individual	20 weeks	Home	1 h	Motor exploration and following a baby’s lead.
10–12	Booster sessions	Individual	20–24 weeks	Home	1 h	Reinforcement in any areas of need.

*NBO refers to the Newborn Behavioural Observations system (Nugent et al., 2007)

The group antenatal sessions focussed on introducing the intervention format and content, building clinical rapport and providing psychoeducation around infant development, in particular social and communication development and infant capacities in these domains. Each postnatal session focussed on a specific theme, building on techniques and learnings from the previous sessions to help parents understand different aspects of their baby’s early communication signals. Video feedback techniques were used in the postnatal sessions and involved filming short interactions between parent and infant in a range of scenarios and activities, which the therapist reviewed to then re-watch with the parent and explore using four feedback tools: (1) “reading the baby” through describing infant’s thoughts, emotions or behaviours by interpreting their physical gestures or emotional expressions; (2) “child development messages” where the therapist provides messages related to the key theme for each session; (3) “enhancement messages” where therapists build on parents’ existing strengths by identifying positive examples of target parent behaviour and encouraging parents to reflect on the effect of the interaction with their infant and (4) “parent coaching moment”, where the clinician encourages the parent to identify what they could do differently through reflective questioning. These feedback tools were used together to build a narrative around the key messages for the session. For example, a session targeting “matching the infant’s pace” may have included opportunities to use “reading the baby” around pace (e.g., “he’s still really focussed on this toy”) or “enhancement messages” when a parent’s pace was well matched to the infant. Each session concluded with a summary discussion to emphasise the key messages, clarify parent understanding, and plan for how to enact these messages in their daily interactions with their infant.

### Measures

Feasibility was assessed across four domains of the framework for conceptualising feasibility studies developed by Bowen et al. (2009): acceptability, implementation, practicality and integration. Acceptability of the intervention was operationalised as the extent to which families viewed the program as suitable and beneficial. This was assessed through a short questionnaire at the conclusion of the antenatal sessions and a questionnaire and semi-structured interview conducted by the study coordinator (J. Granich) at the conclusion of the program which asked parents their views on the intervention, including questions on whether they believed the intervention was beneficial to their family. Implementation was operationalised as the extent to which the program could be fully delivered. It was evaluated through program attendance, as well as interviews with the therapists (AB and LH) at study completion conducted by the study coordinator (J. Granich), who had no involvement in the development or implementation of the intervention and with whom therapists did not have day-to-day interaction. The interview included specific questions to assess whether therapists judged the time commitment of the intervention as feasible to implement and the feasibility of implementing an intervention through parent mediation. Practicality was assessed through feedback from parents through semi-structured interview and through questionnaires, including specific questions around the structure and time commitment of the program, as well as completion of assessments at the treatment endpoint. Integration was assessed through the interview, with specific questions probing whether participants used the learned techniques in everyday life and whether they planned to do so in the future.

#### Infant and Parent Measures

The key aim for this study was a determination of the feasibility of delivering the intervention. Developmental outcomes of the infants were collected when the infants were 6 months of age to establish the feasibility of administering the assessments for the subsequent main trial. Assessment measures collected included Communication and Symbolic Behaviour Scales Developmental Profile Toddler and Infant Checklist (Wetherby & Prizant, 2002), the Autism Observation Scale for Infants (Bryson et al., 2008), eye-tracking, including the familiar-unfamiliar face paradigm (Wagner et al., 2018), the Mullen Scales of Early Learning (Mullen, 1995), and as a measure of parent-child interaction, filming a free-play interaction sample with the elected primary caregiver. However, as this study was implemented to assess the feasibility of the intervention, these are the primary data presented here.

### Data Analyses

Questionnaires collected at the two timepoints (conclusion of the antenatal sessions, and at intervention completion) were assessed using quantitative descriptive analysis of questionnaire responses, and through qualitative analysis of themes identified from qualitative responses in questionnaires. Semi-structured interviews conducted at intervention completion were analysed qualitatively to condense key themes of responses. Attendance was assessed through completion of antenatal and intervention sessions (%), and completion of assessments at the 6-month endpoint was likewise assessed as the number of families that completed these assessments (%). Trustworthiness was addressed throughout the data analysis process to ensure the findings were not the result of preconceived ideas by the authors through triangulation of qualitative themes with findings from quantitative questionnaire data, and repetition and expansion of questions across these formats (Krefting, 1991).

## Results

### Acceptability

Acceptability was assessed through parent questionnaires and semi-structured interviews at the intervention endpoint. Questionnaires assessed the acceptability of antenatal and postnatal sessions through questions examining the logistics, content, and presentation of the intervention. The results of questions related to content and presentation of the intervention are presented in Table 2. Overall, responses indicated that parents agreed or strongly agreed that the antenatal sessions improved their understanding of communication and interactions with infants, and the majority of parents agreed or strongly agreed that these sessions had improved their confidence and readiness for their baby. At the completion of the postnatal sessions, all parents agreed or strongly agreed that they were more comfortable and confident in their parenting and had benefited from taking part in the program.

**Table 2:** Findings from the parent feedback questionnaire

Question	1 (Strongly disagree)	2 (Disagree)	3 (Neutral)	4 (Agree)	5 (Strongly Agree)
Antenatal (*n* = 13)					
I found session 1 to be clear and helpful in introducing the ENGAGE Pilot program.	0 (-)	0 (-)	1 (8%)	5 (38%)	7 (54%)
I understand how early social communication and the environment I create impact on baby’s brain development.	0 (-)	0 (-)	0 (-)	4 (30%)	9 (69%)
I understand that strong connections are formed when I respond thoughtfully to my baby’s cues and needs.	0 (-)	0 (-)	0 (-)	4 (30%)	9 (69%)
I am the most influential person in my baby’s life and my interactions with my baby are powerful for their growing brains.	0 (-)	0 (-)	0 (-)	0 (-)	13 (100%)
I feel ready to get to know my baby’s unique communication cues.	0 (-)	0 (-)	2 (15%)	6 (46%)	5 (38%)
I found session 2 to be clear and helpful in describing when a baby is most ready for interactions *(States of Alertness).*	0 (-)	0 (-)	1 (8%)	7 (54%)	5 (38%)
I know more about how a baby communicates than I did before this session.	0 (-)	0 (-)	0 (-)	6 (46%)	7 (54%)
I found the tips on how babies might communicate with me, and how I might communicate back, helpful.	0 (-)	0 (-)	0 (-)	7 (54%)	6 (46%)
I would like to use the tips on newborn communication with my own baby.	0 (-)	0 (-)	0 (-)	4 (30%)	9 (69%)
I know that at times baby’s cues can be confusing and difficult to interpret.	0 (-)	0 (-)	0 (-)	4 (30%)	9 (69%)
I felt comfortable participating in a group setting.	0 (-)	0 (-)	1 (8%)	10 (77%)	2 (15%)
After completing the antenatal sessions, I feel more confident about communicating with my baby.	0 (-)	0 (-)	2 (15%)	6 (46%)	5 (38%)
I would recommend these antenatal sessions to other parents-to-be to learn about baby cues.	0 (-)	0 (-)	1 (8%)	6 (46%)	6 (46%)
Postnatal (*n* = 11)					
The program has helped me understand more about my baby.	0 (-)	0 (-)	1 (9%)	4 (36%)	6 (54%)
The program taught me skills that I didn’t have previously.	0 (-)	0 (-)	1 (9%)	6 (54%)	4 (36%)
After completing the program, I feel more confident in understanding when my baby is communicating with me.	0 (-)	0 (-)	0 (-)	5 (45%)	6 (55%)
After completing the program, I feel more confident in understanding how to respond to my baby.	0 (-)	0 (-)	0 (-)	6 (55%)	5 (45%)
After completing the program, I feel more confident in my parenting skills.	0 (-)	0 (-)	0 (-)	6 (55%)	5 (45%)
I felt comfortable having these sessions in my own home.	0 (-)	0 (-)	0 (-)	2 (18%)	9 (82%)
I feel that I have benefited from the program.	0 (-)	0 (-)	0 (-)	6 (55%)	5 (45%)
I feel that my infant has benefited from the program.	0 (-)	0 (-)	0 (-)	6 (55%)	5 (45%)
I feel that my family has benefited from the program.	0 (-)	0 (-)	0 (-)	7 (64%)	4 (36%)
I would like to use the skills taught through the program with my baby after the program has finished.	0 (-)	0 (-)	0 (-)	2 (18%)	9 (82%)
I would recommend that other parents undertake this program.	0 (-)	0 (-)	0 (-)	3 (27%)	8 (73%)

Parents reported that the experience of taking part in the intervention had been positive, with the majority reflecting that they had gained awareness and knowledge about infant communication cues; “I’m a lot more aware now and know when and how to slow down when I play with my baby”, and increased knowledge of cues; “I’ve definitely gained knowledge about baby cues and noticed his cues so much more than I would have done otherwise without the intervention which was awesome…when you’re a busy mum it’s easy to just miss all that”. The majority of parents also indicated that video feedback had been beneficial in assisting to notice more about their baby’s development and cues than they would have otherwise; “I really liked the video session to reflect…it allows you to see things you wouldn’t normally see”. Specific suggestions for improvements included reducing the length or number of the antenatal sessions and considering the inclusion of written tip sheets or videos to summarise sessions and revisit or refer to between sessions. One parent reflected that they found group sessions difficult, and two others that they would have enjoyed further contact with the group postnatally. Two parents indicated they would appreciate a follow-up in the months after the intervention ended to check in. Interviews indicated no unwanted or adverse effects of the intervention.

### Implementation

Attendance was 92% for the antenatal session and 100% for the 7 postnatal sessions, with 12/13 (92.3%) families opting in for one or two booster sessions. Therapists reported that the program was logistically feasible to run and to implement through parent mediation. Further reflection through the semi-structured interview from therapists indicated potential improvements on the content and structure of the sessions, including that antenatal sessions could be combined into one session, in particular given an awareness of the considerable demands on parents’ time during the third trimester. For postnatal sessions, logistical challenges reported included the difficulties of working with babies, meaning that session times could coincide with the baby needing to feed or sleep, and a reflection on the importance of maintaining flexibility in the delivery of this program. Reflection on the process of session delivery also indicated the potential need for a more flexible delivery of teachings for parents who are less reflective or who might struggle with introspection.

### Practicality

After the antenatal sessions, the majority of parents indicated the sessions were the correct length, and number, and all agreed that they were run at an appropriate time in their pregnancy. At the end of the postnatal intervention, all parents agreed that the sessions were the correct length, number and an acceptable time commitment. Parents additionally reflected that the home-based setting made the intervention practical, “especially when you have a new baby, and you don’t know what day or time it is”. Infant developmental measures at the intervention endpoint were conducted for 9 of 13 families, with four families not completing the final infant in-person assessments due to reservations around attending assessment during the COVID-19 pandemic, and for 1 family, due to time constraints. Phone interviews were conducted for these families, and assessments collected via post where possible. For those families who were assessed, all assessments were completed, and no difficulties in administering these assessments were encountered. It was noted that flexibility was required to book assessments at a time suitable to the family and infant routine, and flexibility for infant breaks including for feeding during the assessment.

### Integration

All parents agreed or strongly agreed that they would like to continue implementing the skills they had learnt after program completion, with the majority of parents reflecting that they found it straightforward to integrate the program learnings into everyday life interactions with their infant.

### Developmental Outcome Measures

A summary of key infant measures is reported in Table 3. As the key aims of this study were to assess the feasibility of the intervention, no analyses were conducted on these measures, with the practicality of their collection the key outcome as reported above.

**Table 3:** Summary scores from developmental assessments

	*N*	M (SD)	Range
AOSI	9	11.44 (4.88)	4–19
MSEL SS	9	124.56 (12.32)	109–149
CSBS SS	12	108.5 (14.54)	88–135

Note. *AOSI*, Autism Observation Scale for Infants; *MSEL*, Mullen Scales of Early Learning; *CSBS*, Communication and Symbolic Behaviour Scales Developmental Profile Toddler and Infant Checklist; *SS*, Standard Score

## Discussion

This study represents the first description of a theoretically based intervention designed eventually for newborns with an elevated familial likelihood of autism. The study takes the novel step of applying an intervention focussed on parent-infant interactions over the first few months of life, hypothesising that intervening in parent-child interactions during this time may address critical time-bound developmental transitions in vulnerable infants and promote the development of longer-term outcomes. This paper reports feasibility data from a community sample of first-time families.

The key aim of this study was to establish the feasibility of delivering this novel intervention during the perinatal and early infancy time period. Evidence from a variety of sources, including the number of sessions attended, completion of assessments, as well as parental questionnaires and interviews, suggests that this intervention approach (2 antenatal sessions and 7 postnatal sessions over 5 months) and the intervention content were acceptable to families, and able to be integrated into their routines with a newborn. Furthermore, it was found to be feasible in the domains of implementation and practicality. This extends previous research examining the acceptability of interventions of a similar format at later developmental stages (e.g. Green et al., 2013), indicating that a parent-mediated intervention focussed around video feedback is a promising format to be implemented within the perinatal developmental time period.

More detailed feedback addressed in interviews with parents highlighted the importance of minimising the demands of interventions in the perinatal period, with parents showing a preference for shorter or fewer antenatal sessions, which was also reflected by clinicians, who felt that the information could be made more concise, and the time load on parents in their third trimester should be as low as possible. Similarly, the importance of flexibility was highlighted throughout the administration of the program and the importance of incorporating this into the intervention model. Therapist feedback indicated flexibility in the timing of appointments and timing of assessments was required to work around infant routines. Further findings such as parents’ varied experiences with group delivery may point towards the need for interventions to be flexible and individualised further to improve acceptability, with some options for increased group intervention desirable for some parents. However, the convenience and flexibility of home-based sessions remain important for the practicality of an intervention implemented with parents of newborns.

### Limitations and Future Research

Semi-structured interviews allow the collection of a greater depth and breadth of information than a structured interview and allow the participant greater control in the direction of responses; however, these responses are limited by the questions asked and are influenced by the interviewer’s presence and responses (Corbin & Morse, 2003; Diefenbach, 2008). This study used data from questionnaires as well as interview feedback to triangulate parental response data. However, limitations of self-report and interview due to the potential biasing of interviewee responses should be acknowledged.

The key aim of this study was to examine the feasibility of the implementation of this intervention. This study tested the intervention on a community sample—this was essential for the ethics of a neonatal intervention. However, there may be limitations in generalising these results to parents who already have a child presenting with developmental difficulties, where the experience of participating in an intervention such as this one would plausibly be qualitatively and logistically different from the experience of parents having their first child. Parenting a child diagnosed with autism is uniquely challenging, with differences in their child’s development resulting in significant stress on parents (Davis & Carter, 2008; Hastings & Johnson, 2001). Alongside supporting their child’s individual needs within the home and daily life, the recommended therapy for most children diagnosed with autism is time-intense, with the aim of providing a variety of intervention strategies tailored to the individual developmental needs of the child and the family (Myers et al., 2007). Given these challenges, it would be expected that having an autistic child influences the experience of parenting, and as such the experience of parenting subsequent children, however, literature in this area is scarce.

Of additional consideration is the impact of birth order on parent availability to engage with an intervention. The sample used here being a sample of first-time parents is different from that of families who already have a child diagnosed with autism, where infants involved in the intervention would be second or further in birth order. Research provides some indication that, overall, first born children receive more time from parents than subsequent children (Bornstein et al., 2019); however, for infant siblings of autistic children, it is difficult to ascertain whether this is also found. While parental engagement is clearly identified as an important factor in mediating the success of parent-mediated interventions broadly (Piotrowska et al., 2017), the impact of birth order, child diagnostic status and other individual family factors of consideration require further exploration. Additionally, further exploration of the acceptability of this intervention across different ethnic and cultural groups will help determine whether the intervention may be able to be adapted for broader population groups.

While these considerations limit the interpretation of our findings, this study provides a positive initial indication of the feasibility of this intervention in a community sample. While this intervention was designed for infant siblings of autistic children, it is adapted from an intervention with proven efficacy for improving parent-infant interaction, parental responsiveness and infant attachment in neurotypical populations (Juffer et al., 2008; Moss et al., 2011; Stein et al., 2006). We therefore consider there is prima-facie evidence of the appropriateness and potential efficacy of this intervention in the context where many recipients will turn out to be neurotypical. Future research could extend the current findings through exploring the experiences of parents of autistic children to further refine the design of this intervention.

We have presented here the theoretical rationale for very early interventions commencing in the perinatal time period and targeting interactions in the first months of infancy. Future examination of the efficacy of this intervention is required, to establish the impact on parent-child interactions as well as child development. It is hypothesised, following the findings of this study alongside previously implemented interventions, that the implementation of this program would be found to be feasible to implement with families with infants at elevated likelihood of autism. Furthermore, following the developmental science rationale presented here, we hypothesise that the implementation of this intervention with a group of infants at elevated likelihood of autism would improve social and communication outcomes.
